# Epigenetics in cancer stem cells

**DOI:** 10.1186/s12943-017-0596-9

**Published:** 2017-02-01

**Authors:** Tan Boon Toh, Jhin Jieh Lim, Edward Kai-Hua Chow

**Affiliations:** 1 Cancer Science Institute of Singapore, National University of Singapore, Singapore, Singapore; 2 Department of Pharmacology, Yong Loo Lin School of Medicine, National University of Singapore, Singapore, Singapore; 3 Centre for Translational Medicine, National University of Singapore, 14 Medical Drive #12-01, Singapore, 117599 Singapore

**Keywords:** Cancer stem cells, Epigenetics, Histone methylation, Histone methyltransferase, DNA methylation, Signaling pathway

## Abstract

Compelling evidence have demonstrated that bulk tumors can arise from a unique subset of cells commonly termed “cancer stem cells” that has been proposed to be a strong driving force of tumorigenesis and a key mechanism of therapeutic resistance. Recent advances in epigenomics have illuminated key mechanisms by which epigenetic regulation contribute to cancer progression. In this review, we present a discussion of how deregulation of various epigenetic pathways can contribute to cancer initiation and tumorigenesis, particularly with respect to maintenance and survival of cancer stem cells. This information, together with several promising clinical and preclinical trials of epigenetic modulating drugs, offer new possibilities for targeting cancer stem cells as well as improving cancer therapy overall.

## Background

Advances in genomic and epigenomic research has shaped our understanding of cancer over the past two decades. Rather than merely a perpetuating mass of dysregulated cells growing in an uncontrolled manner, cancer is also defined by the dynamic genetic and epigenetic alterations that contribute to cancer initiation and progression. Since epigenetic changes such as DNA methylation and histone modifications are crucial factors in developmental programming of stem cells to specific lineages of cellular and tissue differentiation, aberrant epigenetic alterations may transform normal stem cells to cancer stem cells with the loss of differentiation capacity and the acquisition of stem-like characteristics. More importantly, epigenetic mechanisms have been shown to be implicated in the observed variability of treatment response. For instance, a small subset of cells has been shown to be resistant to drug therapy in a variety of cancers such as melanoma, gastric, colon and lung cancers as a result of aberrant expression of key epigenetic modifiers. In this review, we will focus our discussion on the epigenetic regulation of CSCs and their impact on tumor-initiation, progression and response to therapies. We will also discuss recent advances in using epigenetic therapy to target cancer stem cells.

## Main text

### Cancer stem cells (CSCs)

Cancer stem cells (CSCs) define a small, unique subset of cells with self-renewal ability and the capacity to generate the different cell types that constitute the whole tumor [1]. These cells are termed CSCs because of their “stem-like” properties commonly shared with normal tissue stem cells. Such properties include extensive self-renewal ability (symmetrical and asymmetrical) and differentiation capacity. It should be noted that a general capacity to differentiate is not a mandatory feature of CSCs and that the ability of CSCs to differentiate and repopulate the cell types found in the original tumor is of greater significance. More importantly, CSCs should demonstrate potent tumor-initiation capacity. This property is usually demonstrated by injecting limited number of CSCs into an orthotopic in vivo environment to generate the bulk tumor. Nevertheless, the concept of CSC is of significant importance as it highlights the need to eradicate the CSC populations to achieve an effective cure.

The first clear evidence of CSCs being a key tumor-initiating subset of cancer cells was demonstrated in acute myeloid leukemia (AML) where prospective CSCs were isolated using cell surface markers that identify normal haematopoietic stem cells and evaluated for their tumor-initiating properties [2, 3]. Since then, similar identifications of tumor-initiating populations have been identified in multiple solid tumors that includes brain, breast, liver, ovary, prostate, lung, melanoma and colon cancers, by using different cell surface markers or through side population (SP) analysis [4–11]. For instance, in malignant glioma and medulloblastoma tumors, a putative neural stem cell marker, CD133 has been demonstrated to be adequate and essential to initiate and recapitulate the tumor upon xenotransplantation into immune-compromised mice [5]. However, this finding has been disputed as tumors can also arise from CD133-negative cells in a subset of glioma tumors [12]. In addition, CD133 surface marker expression has been demonstrated to change according to disease state and progression, further complicating its role as a *bona fide* CSC marker in brain tumors [13, 14]. In liver cancers such as hepatocellular carcinoma (HCC) and intrahepatic cholangiocarcinoma (ICC), similar use of cell surface markers such as epithelial cell adhesion molecule (EpCAM), cytokeratin 19 (CK19), CD133, CD90, CD44, CD24, and CD13 has been applied to define a subpopulation of liver cancer cells as CSCs [15]. Importantly, it has recently been shown that these CSC markers are not specific to liver CSCs, and that distinct populations of liver CSCs express different surface markers possibly due to the strong intra- and inter-heterogeneity and varied etiology of liver cancer [16]. As a result, CSC studies have begun to move away from the reliance of cell surface markers to identify tumor-initiating cells and have begun to identify other complementary methods of measuring the functional activities of CSCs that may serve to identify CSCs as well as the molecular mechanisms that regulate CSCs [17].

Currently, the central theme of the CSC model is the ability of a subset of cells at the apex of the hierarchy to propagate tumors and promote tumor progression as compared to the non-tumorigenic cells within the bulk tumor. One of the gold standards to functionally identify CSCs is the capacity of these cells to regenerate a phenotypic copy of the original tumor in an orthotopic transplantation model. Non-CSCs, by definition, lack this ability and fail to generate tumors in the transplantation model. It is important to note that the CSC hierarchy model may not be ubiquitous for all cancers and that some tumorigenic cells are common in certain cancers. It is also important to note that such transplantation assays measure the tumorigenic potential of the cells to form tumors and not their actual fate. For example, alterations in tumorigenic assays carried out by Quintana and colleagues showed that CSC frequency could be increased by changing several experimental parameters such as the use of extracellular matrix (ECM) in the form of matrigel, prolonging the duration for tumor formation, and varying the severity of immune-compromised mice used [18]. This study highlighted that the tumor-initiating capacity may be an artificial consequence of the conditions employed in xenograft mouse models.

While analyzing CSC surface marker expression in primary tumors has been often performed to study the clinical impact of CSCs on tumor progression, more often than not, this has resulted in ambiguous data possibly due to the fact that CSC properties that sustain the primary tumor phenotype are defined by more than just specific marker expression [19, 20]. Analysis of key signalling pathway activity that resembles those functioning in stem-like cells, is more likely to accurately interrogate the clinical contribution of CSCs. An example of such studies was carried out by Lim et al. in *BRCA1* mutation-associated breast tumors, where the authors prospectively isolated distinct subpopulations of normal and tumorigenic epithelial cells from BRCA1 mutation heterozygous individuals and found that luminal progenitors were highly represented in *BRCA1* mutation-associated breast tumors, more than the stem cell population [21]. This suggests that luminal progenitors are more likely the cells-of-origin for BRCA1 mutation-associated breast tumors, which was later confirmed in a transgenic mouse model study carried out by Molyneux and colleagues [22]. These studies highlight the predictive capability of gene expression mapping of pathway activation rather than specific marker identity. In a separate study, John Dick and colleagues demonstrated that tumor-initiating AML stem cells contribute to disease progression and patient survival outcome, underscoring the importance of functionally defining the CSCs [23]. More importantly, the contribution of CSCs, with preferential activation of core stem cell programs, to patient survival outcome has been demonstrated. The study by Shats et al. showed that a stemness gene signature derived from embryonic stem cells (ESCs) could predict a breast cancer patient cohort sensitive to drugs linked to this signature using a Connectivity Map [24], demonstrating the clinical contribution of CSCs to patient outcome [25]. Collectively, these studies highlight that CSCs that perpetuate tumors are not merely defined by surface marker expression, but more importantly and more accurately by their gene expression profiles and consequent pathway activations.

### Epigenetics: normal and cancer stem cells

Epigenetic regulation of the genome is one of the primary means by which genetic code is altered to control cellular developmental hierarchies. Epigenetic mechanisms such as histone modifications, DNA methylation, chromatin remodelling and even changes in noncoding RNAs including miRNAs together govern the epigenome landscape that dictate the outcome of cell fate specification without changes to the DNA sequences. Such changes in the genome is important during normal mammalian development and ESCs differentiation [26]. Importantly, gene expression profiles change during cellular differentiation according to not only a network of transcription factors but also the “epigenomic landscape” of the cell. For the purpose of this review, we will focus our discussions on two primary mechanisms of epigenetic regulation: histone modifications and DNA methylation.

Histone methylation occurs predominantly on lysine (K) and arginine (R) residues and these methylation marks serve as docking sites for histone readers [27]. Both lysine and arginine methylation can occur on both histones and non-histone proteins. The highly conserved histone lysine methylation occurs at three different levels: mono-, di-, and tri-methylation. Such modifications are commonly associated with gene activation or repression, depending on the target histone modification. For instance, histone H3 lysine 4 (H3K4), histone H3 lysine 36 (H3K36), and histone H3 lysine 79 (H3K79) are associated with gene activation whereas histone H3 lysine 9 (H3K9), histone H3 lysine 27 (H3K27) and histone H4 lysine 20 (H4K20) are associated with gene repression. The N-terminal tails of histones frequently undergo other post-translational modifications, which play significant roles in various DNA-templated processes, including transcription [28]. Hence, aberrations in histone modifications can lead to deregulated gene expression as seen in various human disease and malignancies.

DNA methyltransferases (DNMTs) are a class of enzymes involved in transferring a methyl group from S-adenosyl methionine (SAM) to cytosine bases of CpG dinucleotides at gene promoters and regulatory regions [29]. CpG dinucleotides are concentrated in short CpG-rich regions known commonly as “CpG islands”. In humans, CpG islands occupy about 60% of the gene promoters. CpG promoter islands can be methylated during development that results in long-term gene silencing. One classic example of such naturally occurring CpG methylation is the X-chromosome inactivation and the imprinted genes. DNA hypermethylation has also been associated with the silencing of tumor suppressor genes as well as differentiation genes in various cancers [30]. The reduced expression of these genes may then contribute to the formation of CSCs within tumor cell populations [31, 32]. Indeed, the importance of DNA methylation in maintaining CSC properties have been reported in leukemic, lung and colon stem cells [33–35]. The accumulation of epigenetic abnormalities has been suggested to be an early event that predisposes these tumor cells to acquire further mutations and genomic instability. This is supported by the fact that epigenetic machinery is crucial for the maintenance of normal stem and progenitor cells and that any epigenetic deregulation can lead to accumulation of cells with increased stemness properties and self-renewal ability, thus giving rise to CSCs.

### Key CSC pathways regulated by epigenetic mechanisms

#### Wnt/β-catenin signaling pathway

The canonical Wnt/β-catenin signaling pathway mediates gene activation through the transcription factor β-catenin. In the absence of Wnt signaling, cytoplasmic β-catenin is inactivated by a degradation complex comprising Adenomatous polyposis coli (APC), Axin, glycogen synthase kinase 3 beta (GSK-3β), and casein kinase 1 (CK1). Phosphorylation by GSK-3β targets β-catenin for ubiquitination and subsequent proteasomal degradation. Upon Wnt ligand binding to Frizzled receptors, the degradation complex is inactivated via low density lipoprotein receptor-related protein 5/6 (LDR5/6) and Dishevelled, allowing for stabilisation of β-catenin. Accumulated β-catenin then translocates into the nucleus, where it associates with T-cell factor/lymphoid enhancer factor (TCF/LEF) transcription factors to induce transcription of Wnt target genes such as *CCND1* and *MYC*. The Wnt/β-catenin pathway has important functions in normal tissue development and maintenance, as well as in self-renewal and differentiation of CSCs [36, 37]. In fact, the Wnt/β-catenin pathway has been found to be aberrantly activated in a variety of cancers, either via genetic alterations, such as mutations in *CTNNB1, APC* and *AXIN* genes [38–40], or through epigenetic modulation.

**Fig. 1:**
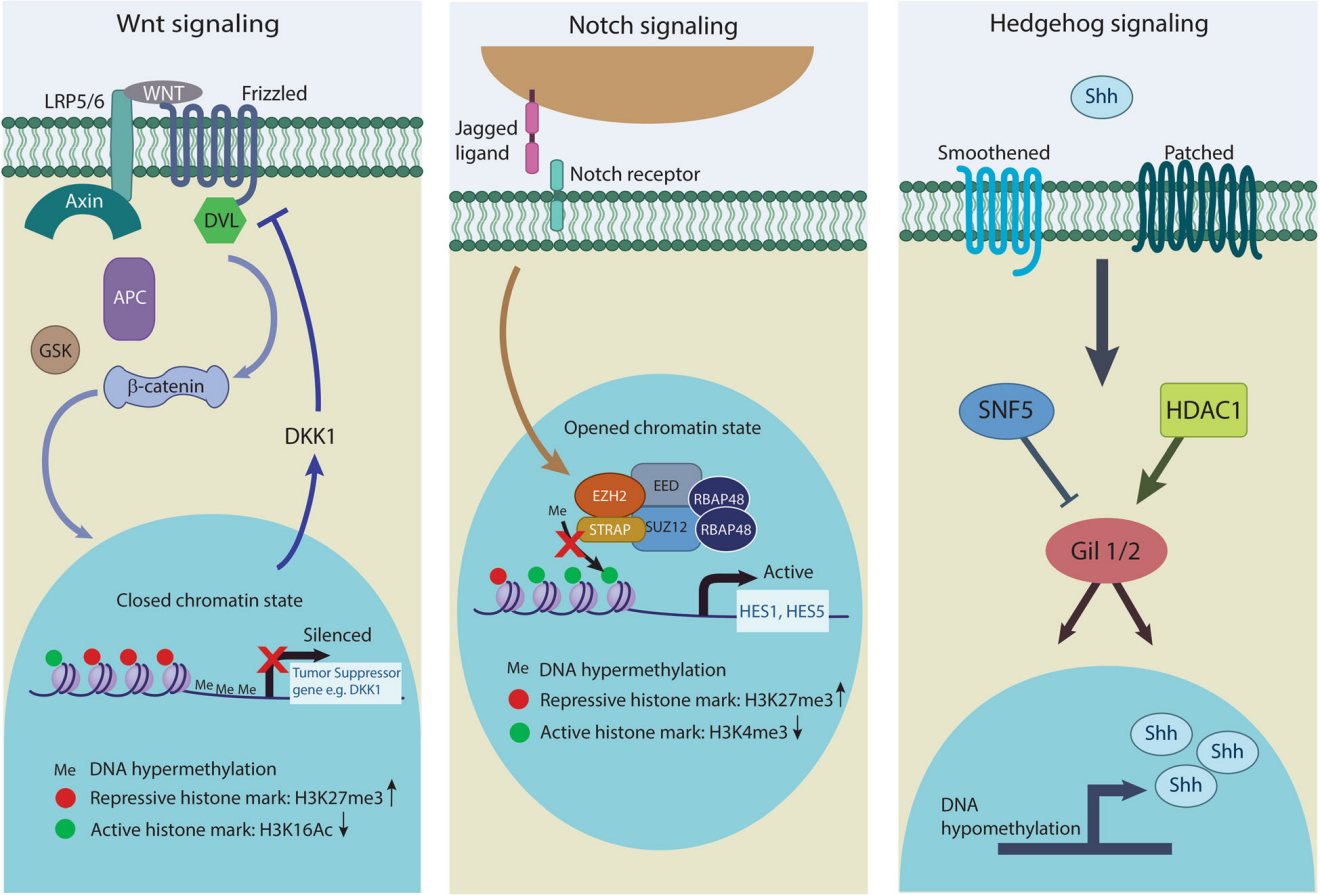
Regulation of key cancer stem cell signaling pathways by epigenetic mechanisms. Wnt/β-catenin signaling can be enhanced by decreased expression of the DKK1 inhibitor through promoter hypermethylation and increased H3K27me3 and decreased H3K16 acetylation marks. Notch signaling target genes such as Hes1 and Hes5 can be activated by inhibition of H3K27 inhibitory methylation mark at their promoter region by STRAP. Hedgehog signaling pathway can be activated in CSCs epigenetically by Shh promoter hypomethylation and increase HDAC1 expression. Epigenetic deregulation of CSC-related signaling pathways allows cancer cells to acquire self-renewal ability and drug resistance properties

DNA methylation has been linked to aberrant Wnt/β-catenin pathway activation through the enhanced promoter methylation and subsequent silencing of various Wnt inhibitors such as Wnt inhibitory factor 1 *(WIF-1), AXIN2,* Secreted frizzled-related protein 1 *(SFRP-1),* and Dickkopf-related protein 1 *(DKK1)* in breast and colorectal cancers [41–43]. In gastric cancer, Yoda et al. showed that aberrant methylation of Wnt negative regulators, including *DKK3,* Naked cuticle homolog 1 *(NKD1)* and *SFRP1*, could lead to activation of Wnt/β-catenin pathway [44]. Deregulation of Wnt/β-catenin pathway in cancer is also mediated by aberrant histone modifications. Decreased acetylation of H3K16 and increased H3K27 trimethylation along with recruitment of Sirtuin 1 (SirT1), enhancer of zeste homolog 2 (EZH2) and suppressor of zeste 12 protein homolog (Suz12) (components of polycomb repressor complex 2, PCR2) to the promoter of DKK1 inhibited the expression of the DKK1 Wnt antagonist (Fig. 1) [45]. In colorectal cancer, Dishevelled-binding antagonist of beta-catenin 3 (DACT3), an antagonist of Dishevelled, was found to be regulated by bivalent histone modifications—activating H3K4me3 and repressive H3K27me3 histone marks—at its locus [46]. This bivalent histone state was associated with decreased DACT3 expression in colorectal cancer cell lines [46]. In addition, methylation of H3K4 at the regulatory element of DKK1 marks the site for binding by the transcription factor Achaete-scute family BHLH transcription factor 1 (ASCL1), resulting in a repressed chromatin configuration [47]. ASCL1-mediated inhibition of DKK1 consequently leads to activation of Wnt signaling, and ASCL1 was found to be crucial for glioblastoma CSC maintenance and tumorigenicity [47–49].

Besides alterations in DNA and histones, non-coding RNAs have also been found to act as epigenetic modulators of Wnt/β-catenin signaling. Wang et al. demonstrated that long non-coding RNA of transcription factor 7 *(lncTCF7)*, which is highly upregulated in liver CSCs, is able to induce TCF7 expression by recruiting the Switch/sucrose non-fermentable (SWI/SNF) chromatin remodelling complex to its promoter [50]. This subsequently activates the Wnt pathway, leading to self-renewal of liver CSCs and tumor propagation.

#### Hedgehog signaling pathway

The Hedgehog (Hh) signaling pathway plays important roles in guiding cell fate during embryonic development and in maintaining adult tissue homeostasis [51, 52]. It also functions in regulating stem and progenitor cell proliferation and maintenance in several tissues [53]. In the absence of sonic hedgehog ligand (Shh), the Patched receptor (PTCH1) prevents activation of Smoothened (SMO), allowing Gli proteins to be sequestered by suppressor of fused homolog (SUFU) and kinesin family member 7 (Kif7). Upon Shh binding to PTCH1, SMO is activated and mediates Hh signaling transduction via release of Gli proteins, which then enter the nucleus and act as transcription factors. Gli1 activates transcription of Hh target genes, Gli2 can both activate and repress transcription, while Gli3 functions as a transcriptional repressor.

The Hh signaling has been implicated in tumorigenesis in various tissues [54]. In basal cell carcinoma (BCC), upregulation of Hh signaling in stem cells in the interfollicular epidermis [55] or within the hair follicle [56] has been reported to contribute to tumor formation. In medulloblastomas, granule neuron progenitors/precursors (GNPs) in the cerebellum that have constitutively active Hh signaling have been identified as cells-of-origin of the tumor [54, 57, 58].

The Hh pathway is activated by genetic mutations in both BCC and medulloblastoma. However, epigenetic mechanisms also play a role in modulating the expression and function of Hh pathway components in various tumors. The chromatin remodelling protein SNF5 directly interacts with Hh signaling effector Gli1 to downregulate expression of Hh target genes. SNF5 is a member of the SWI-SNF complex and inhibits gene expression by altering chromatin structure at Gli1-regulated promoters, which includes genes such as Ptch1 and Gli1 itself. Hence, inactivation of SNF5 would contribute to aberrant Hh signaling activity as seen in human malignant rhabdoid tumors [59–61].

In addition, histone deacetylases are also involved in regulating Gli protein function. Gli1 and Gli2 proteins require deacetylation by HDAC1 to be transcriptionally active, and they, in turn, can induce HDAC1 expression through a positive autoregulatory loop [62]. This mechanism is inhibited by E3-ubiquitin ligase complex (comprising Cullin3 and renin, REN)-mediated degradation of HDAC1. However, *REN* is often deleted in human medulloblastoma [63], resulting in increased levels of HDAC1 and Gli1, and subsequent deregulation of Hh signaling in neural progenitors and tumor cells [62].

Hh pathway can also be epigenetically regulated by aberrant DNA methylation. Studies have found that hypomethylation of Shh promoter leads to enhanced expression of Shh ligand in breast and gastric cancers (Fig. 1) [64, 65]. Indeed, Duan and colleagues reported that promoter hypomethylation allowed nuclear factor kappa b (NF-κB) to bind and activate transcription of Shh, resulting in overexpression of the ligand [66]. Consequently, the upregulation of Hh signaling was able to promote self-renewal and invasiveness in breast cancer cells [66].

#### Notch signaling pathway

Notch is a transmembrane receptor involved in cell contact-dependent signaling [67]. Binding of ligands Jagged1/2 or Delta1-4 triggers cleavage of Notch intracellular domain (NICD) by γ-secretase and its release into the cytoplasm [68]. NICD then translocates into the nucleus, where it interacts with recombination signal binding protein for immunoglobulin kappa J region (RBPJ-κ) to transcriptionally induce expression of Notch target genes, such as *MYC* and *HES1* [69]. In the inactive state, RBPJ-κ recruits co-repressor complexes to suppress Notch target genes [70].

Notch signaling is an evolutionarily conserved pathway that has important roles in development of various tissues and organs [71]. It also regulates cell proliferation and differentiation across a wide range of cell types and during different stages of cell lineage progression [69]. Furthermore, Notch pathway modulates stem cell differentiation and self-renewal. Importantly, Notch signaling has been shown to be crucial for survival of neural stem cells (NSCs) [72]. In murine intestinal stem cells, loss of B-lymphoma Mo-MLV insertion region 1 homolog (Bmi1), a target of Notch signaling, decreases proliferation and induces cellular differentiation into goblet cells [73]. Deregulation of Notch pathway has been implicated in various tumors such as prostate cancer, breast cancer, lung cancer, colorectal cancer and haematological malignancies [74–77]. Recent studies have also reported the role of Notch signaling in breast, colon and oesophageal CSCs [78–80].

Epigenetic modifications affecting various components of the Notch pathway have been found to cause aberrations in Notch signaling activity. Overexpression of Notch ligand Jagged2 in multiple myeloma has been associated with enhanced histone acetylation at the *JAGGED2* promoter region [81]. Nuclear co-repressors such as nuclear receptor co-repressor 2 (SMRT) normally recruit HDACs to promoter regions to regulate gene expression. However, in multiple myeloma, the decreased levels of nuclear co-repressor SMRT reduces HDAC recruitment to *JAGGED2* promoter, resulting in increased transcription of the Notch ligand and subsequent activation of Notch signaling [81]. In addition, Jin et al. reported that serine-threonine kinase receptor-associated protein (STRAP) promotes stemness in colorectal cancer-initiating cells via modulating the Notch pathway [80]. They found that STRAP interacts with EZH2 and SUZ12 of PRC2 complex, inhibiting histone methylation of H3K27 on *HES1* and *HES5* promoters, leading to gene activation (Fig. 1). This was concordant with the finding that both genes had increased activating (H3K4me3) and decreased repressive (H3K27me3) histone marks in wild-type (WT) cells as compared to STRAP knockdown (KD) cells. Moreover, ectopically expressed *HES1* or *HES5* was able to rescue the stemness phenotype in STRAP KD cells [80], further demonstrating the significance of Notch signaling in regulating stemness potential in CSCs.

#### Epigenetic regulation of metastasis and chemoresistance pathways

During tumor progression, metastasis of tumor cells has been linked to the induction of epithelial-to-mesenchymal transition (EMT). EMT is a multi-step process that results in decreased cell-cell adhesion, loss of cell polarity, increased cell motility, and gain of invasive mesenchymal properties [82, 83]. There is evidence that activation of EMT can confer cells with CSC and tumor-initiating properties [84, 85]. It was reported that EMT induction in both immortalised and transformed human mammary epithelial cells resulted in increased expression of CSC markers and mammosphere formation. Moreover, stem-like cells of mammary carcinomas were also found to express markers of EMT [85]. The relationship between EMT and acquisition of stem-like properties in tumor cells suggests that stemness properties may help increase the chances of disseminated tumor cells to successfully metastasize to distant sites [70].

Several signaling pathways involved in embryonic development, such as Wnt, Hedgehog and Notch, have been identified to regulate the EMT process [86, 87]. The transforming growth factor-β (TGF-β) family of cytokines are also known inducers of EMT [88, 89]. Hence, deregulation of these pathways and proteins could activate aberrant EMT induction, resulting in tumor metastasis and contribute to poorer patient prognosis. A hallmark of EMT is the loss of membrane protein E-cadherin, which functions in maintaining cell-cell adhesion [90–92]. Loss of E-cadherin can arise from mutations in its encoding gene *CDH1*, or via mechanisms that regulate its expression and function, including transcriptional repressors Twist-related protein 1 (TWIST1), Snail family zinc finger 1 (SNAIL), Zinc finger E-box-binding homeobox 1 (ZEB1) and Zinc finger E-box-binding homeobox 2 (ZEB2) [93]. Epigenetic mechanisms have also been found to play a dynamic role in silencing E-cadherin expression. For instance, DNA methylation of E-cadherin promoter helps to recruit HDACs to the site, leading to histone deacetylation and transcriptional silencing [94, 95]. In addition, histone methylation of *CDH1* promoter by EZH2 and PRC2 complex, which is recruited by Snail1, also represses E-cadherin expression [96, 97].

Micro RNAs (miRNAs) that regulate the EMT pathway are epigenetically regulated as well. MiR-200 family members and miR-205 repress EMT and invasion by directly inhibiting transcription factors ZEB1 and ZEB2 [98–100]. Hence, inhibition of these miRNAs would result in increased EMT and metastasis. This is observed in high-grade breast cancers, whereby low levels of miR-200c is correlated with upregulation of EMT and stemness markers [101]. Silencing of miR-200c and miR-205 expression can also occur via enrichment of H3K27me3-mediated chromatin remodelling and DNA methylation, which leads to induction of EMT and CSC phenotype in immortalised human bronchial epithelial cells [102].

Studies have shown that cells with both CSC properties and EMT-like phenotype tend to be more resistant to chemotherapy drugs as compared to other cancer cell populations [103–105]. Arumugam et al. demonstrated that pancreatic cancer cell lines with EMT features were resistant to common chemotherapy drugs such as gemcitabine, 5-fluorouracil and cisplatin [106]. Moreover, cells that were resistant to gemcitabine expressed high ZEB1 and low E-cadherin, and acquired greater cell migration ability [106]. Indeed, these findings indicate that epigenetic modulations involved in the gain of CSC and EMT properties would most likely impact tumor cells’ response to therapy.

The increased drug resistance observed in CSCs is commonly mediated by enhanced expression of drug efflux transporters, such as ATP-binding cassette (ABC) family of transporters, which includes ATP-binding cassette sub-family G member 2 (ABCG2), multidrug resistance protein 1 (MDR1) and multidrug resistance-associated protein 1 (MRP1) [17, 107, 108]. These drug transporters utilise ATP in moving drugs out of the cell against its concentration gradient. The expression of these transporters are regulated by various mechanisms and pathways, and their deregulation would result in an enrichment of these proteins and drug efflux capability. Studies have shown that MRP1 expression can be upregulated by Notch signaling, and is responsible for drug resistance in CSCs [109, 110]. Expression of ABCG2 is upregulated upon enrichment of permissive histone modifications such as greater histone H3 acetylation, increased H3K4 tri-methylation and phosphorylation of H3S10, as well as decreased HDAC1 levels [111]. These histone marks along with decreased H3K9 tri-methylation allow RNA polymerase II and chromatin remodelling protein Brahma-related gene 1 (Brg1) to gain access to the promoter and activate transcription of ABCG2 [111]. Collectively, a complex network of signaling pathways that function in modulating the activity of normal stem cells can be susceptible to deregulation as a result of aberrant epigenetic modifications during the course of tumor formation. These abnormal alterations in key signaling pathways contribute to CSC proliferation and maintenance, as well as tumor progression and invasion. Hence, epigenetic regulation of these signalling pathways may serve as potential mechanisms for targeted therapy against CSCs.

### Therapeutic intervention using epigenetic modifying drugs

As epigenetic mechanisms have important functions in modulating stem cell properties in cancer cells, targeting components of these epigenetic pathways would help in eradicating both CSCs and the bulk tumor population. Inhibitors of epigenetic modulatory enzymes such as HDACs and DNMTs have been widely studied and many are currently in clinical trials for treatment of a variety of cancers. In addition, deregulation of chromatin remodelling has been associated with tumorigenesis and tumor progression, thus making chromatin remodelling proteins viable targets for small molecule inhibitors as well. Indeed, many of these therapeutic strategies aim to induce differentiation of CSCs and to sensitise these cells to chemotherapy, with the ultimate goal of reducing tumor relapse and improving patient survival. Here, we review the development of various epigenetic therapies designed to target different components of the epigenetic machinery. A summary of these epigenetic drugs and their clinical status can be found in Table 1.

**Table 1:** Epigenetic modulators in cancer

Drug	Target	Clinical status	Indication	References
DNMT inhibitors				
Azacitidine	Inhibit DNMT (act as nucleoside analog)	FDA-approved	MDS	[277, 278]
Decitabine	Inhibit DNMT (act as nucleoside analog)	FDA-approved	MDS	[115, 277]
SGI-110	Inhibit DMNT by incorporating into guanine nucleotide	Phase 3 (NCT02348489 Phase 1/2 (NCT01261312, NCT02197676) Phase 2 (NCT01752933) Phase 1/2 (NCT01696032)	AML MDS, AML Advanced HCC Platinum-resistant recurrent ovarian cancer	[279–281]
HDAC inhibitors				
Vorinostat	Inhibitor of Class I and II HDACs	FDA-approved	Cutaneous T cell lymphoma	[135]
Romidepsin	Inhibitor of Class I HDACs	FDA-approved	Cutaneous T cell lymphoma	[136]
Panobinostat	Pan-HDAC inhibitor	Phase 3 (NCT01034163) Phase 2/3 (NCT00425555)	Hodgkin’s lymphoma Cutaneous T cell lymphoma	[282, 283]
Entinostat	Inhibitor of Class I HDACs	Phase 2 (NCT00866333) Phase 1/2 (NCT01038778)	Hodgkin’s lymphoma Clear cell renal cell carcinoma, metastatic renal cell cancer	[284]
Belinostat	Inhibitor of Class I and II HDACs	Phase 2 (NCT00357032) Phase 2 (NCT00274651) Phase 2 (NCT00301756)	Relapsed/refractory AML or older patients with newly diagnosed AML Recurrent/refractory cutaneous and peripheral T cell lymphomas Ovarian cancer	[285–287]
Pracinostat	Inhibitor of Class I and II HDACs	Phase 2 (NCT01112384, NCT01075308)	Translocation-associated recurrent/metastatic sarcomas, metastatic prostate cancer	[288, 289]
Givinostat	Inhibitor of Class I and II HDACs	Phase 2 (NCT01761968)	Chronic myeloproliferative neoplasms	[290]
Valproic acid	Inhibitor of Class I and II HDACs	Phase 2 (NCT01900730)	Breast cancer	[163, 291]
HMT inhibitors				
EPZ-5676	Inhibit DOT1L methyltransferase (H3K79) activity by competing with SAM	Phase 1 (NCT02141828, NCT01684150)	MLL-fusion leukemia, AML, acute lymphocytic/lymphoblastic leukemia, MDS, myeloproliferative disorders	[182]
DZNep	Inhibit HMT activity of EZH2 via inhibiting S-adenosylhomocysteine (SAH) hydrolase	Not in trial	Breast cancer, prostate cancer, glioblastoma multiforme (GBM)	[193, 292, 293]
E7438 (EPZ-6438)	Inhibit HMT activity of EZH2 by competing with co-factor S-adenosyl-methionine (SAM)	Phase 2 (NCT02860286, NCT02601950)	Malignant mesothelioma, rhabdoid tumors, synovial sarcoma, epitheloid sarcoma	[196]
GSK2816126 (GSK126)	Inhibit HMT activity of EZH2 by competing with co-factor S-adenosyl-methionine (SAM)	Phase 1 (NCT02082977)	Relapsed/refractory DLBCL, transformed follicular lymphoma, multiple myeloma, non-Hodgkin’s lymphoma, solid tumors	[198, 199]
CPI-1205	Inhibit HMT activity of EZH2 by competing with co-factor S-adenosyl-methionine (SAM)	Phase 1 (NCT02395601)	B-cell lymphoma	[294]
Chaetocin	Inhibit SUV39H1	Not in trial	HCC, multiple myeloma	[216, 218]
BIX01294	Inhibit G9a (substrate-competitive)	Not in trial	Breast cancer, colon cancer	[209, 212]
UNC0638	Inhibit G9a (substrate-competitive)	Preclinical	Breast cancer	[213]
UNC0642	Inhibit G9a (substrate-competitive)	Preclinical	Pancreatic cancer	[214]
HDM inhibitors				
Tranylcypromine	Irreversible inhibitor of LSD1	Phase 1 (NCT02273102, NCT02717884) Phase1/2 (NCT02261779)	AML, MDS AML	[226]
ORY-1001	Irreversible inhibitor of LSD1	Phase 1	AML	[227]
GSK2879552	Irreversible inhibition of LSD1 activity by modifying its cofactor FAD	Phase 1 (NCT02034123, NCT02177812)	Small cell lung cancer, AML	[231]
GSK-J4	Inhibit UTX	Not in trial	T-cell acute lymphoblastic leukemia (T-ALL)	[295]
BET inhibitors				
I-BET762 (GSK525762A)	Interfere with binding of BET proteins to acetylated histones	Phase 1 (NCT01587703, NCT01943851)	Solid tumors, relapsed refractory haematological malignancies	[243, 244]
JQ1	Interfere with binding of BET proteins to acetylated histones (greatest specificity for BRD3 and BRD4)	Not in trial	NUT midline carcinoma (NMC), multiple myeloma, AML, Burkitt’s lymphoma, DLBCL	[239, 240, 296, 297]
I-BET151 (GSK1210151A)	Interfere with binding of BET proteins to acetylated histones	Not in trial	MLL fusion leukemia, medulloblastoma	[298, 299]
OTX015	Interfere with binding of BET proteins to acetylated histones	Phase 1 (NCT01713582)	Hematological malignancies	[248, 297]
CPI-203	Interfere with binding of BET proteins to acetylated histones	Not in trial	Lymphoma	[300]
CPI-0610	Interfere with binding of BET proteins to acetylated histones	Phase 1 (NCT02157636, NCT01949883, NCT02158858)	Acute leukaemia, MDS, myelodysplastic/myeloproliferative neoplasms, lymphoma, multiple myeloma	[301]

#### Targeting DNA methylation - DNA methyltransferase (DNMT) inhibitors

Inhibitors of DNA methylation were among the first epigenetic drugs tested for use in treatment of cancer [112]. The most widely studied DNMT inhibitors include azacitidine (5-azacitidine) and decitabine (5-aza-2’-deoxycytidine), which act as analogues of cytosine. These molecules get incorporated into DNA and covalently bond with DNA methyltransferase, thus preventing its function [113, 114] and leading to its degradation [115]. These drugs were initially used as cytotoxic chemotherapeutics in the late 1960s [116], but were found to be highly toxic [117–120]. Subsequently, recent studies have discovered that low doses of DNMT inhibitors had greater efficacy in sustaining decreased DNA methylation and associated re-expression of silenced genes in leukemic and epithelial tumor cells [121]. These lower doses were also able to reduce tumorigenicity and target CSC populations within the tumor. In lung cancer, Liu et al. showed that inhibition of DNMT1 was able to decrease proliferation and tumorigenic ability of lung CSCs [34].

Multiple studies have also demonstrated the role of DNMT inhibitors in differentiation therapy. Pinto et al. showed that azacitidine could induce primary AML cells from patients to differentiate into less or non-malignant cells [122, 123]. Prostate cancer derived-CSCs that were treated with decitabine showed decreased expression of stemness genes Octamer-binding transcription factor 4 (*OCT40029* and Nanog homeobox (*NANOG*), leading to overall reduction in tumor growth [124]. In addition, low doses of SGI-110, a newer DNMT inhibitor, was recently reported to be capable of reprogramming ovarian CSCs to a more differentiated state [125]. Treatment with SGI-110 also decreased tumor-initiating ability and re-sensitized these cells to platinum, suggesting a potential use of DNMT inhibitors in combination with other chemotherapeutic agents in preventing recurrence of ovarian cancer [125]. Both azacitidine and decitabine have been approved by the FDA for treatment of myelodysplastic syndrome (MDS) [126]. Clinical trials for other indications such as AML and colorectal cancer are still ongoing. SGI-110 is also in phases of clinical trials for treatment of various cancers such as AML, MDS, liver cancer and platinum-resistant ovarian cancer.

#### Targeting histone deacetylation - Histone deacetylase(HDAC) inhibitors

An important histone tail modification is acetylation, which is regulated by histone acetyltransferases (HATs) and histone deacetylases (HDACs). HATs are responsible for adding an acetyl group onto lysine residues of histone tail, which neutralizes the positive charge, resulting in a more “open” chromatin structure [127]. In contrast, HDACs remove the additional acetyl group, leading to increased binding affinity between DNA and histones, which is generally associated with gene repression [128]. Very often, deregulated gene silencing in cancers has been associated with aberrant histone deacetylation. For instance, in leukemia, this can be mediated through aberrant recruitment of HDACs by fusion proteins such as Acute myeloid leukemia protein 1 Eight twenty-one protein (AML1-ETO) and Promyelocytic leukemia protein retinoic acid receptor alpha (PML-RARα), which leads to abnormal gene silencing and subsequent leukemogenesis [129, 130]. Besides, HDACs can also acetylate non-histone proteins, including tumor suppressor p53 and oncogene B-cell lymphoma 2 (BCL2), resulting in inhibition of p53-dependent transcription [131] and upregulation of pro-survival protein, BCL2 [132]. Hence, the use of HDAC inhibitors in returning histone acetylation patterns to a normal state has been found to be effective in inducing apoptosis and differentiation as well as inhibit proliferation of tumor cells [129, 133]. These HDAC inhibitors can be divided mainly into two classes—the pan HDAC inhibitors and the class-specific inhibitors [134], and they all function via chelating the zinc atom in the active site of the enzyme [127].

Two HDAC inhibitors, vorinostat (subseroylanilide hydroxamic acid) and romidepsin (depsipeptide), have been approved for treatment of cutaneous T-cell lymphoma [135, 136]. Both drugs were found to produce durable response and efficacy in patients with cutaneous T-cell lymphoma in Phase 2 multi-center trials [135–138]. However, besides cutaneous T-cell lymphoma, monotherapy of vorinostat and romidepsin in treatment of various solid tumors have had little success in clinical trials [139–150]. Apart from these two compounds, many other HDAC inhibitors have also been developed and tested in clinical trials, the details of which have been well-reviewed elsewhere [115, 151–153]. Monotherapies of these compounds, including panobinostat [154, 155], entinostat [156, 157], belinostat [158, 159] and pracinostat (SB939) [160], are being tested against various haematological malignancies and solid tumors.

Another mechanism of action of HDAC inhibitors for cancer treatment is via differentiation or reprogramming of cancer cells. As therapy resistance is a major hurdle in cancer treatment and is often associated with CSCs and epigenetic control [161], HDAC inhibitors possess the ability to induce differentiation of CSCs from their quiescent state, thereby re-sensitising them to other chemotherapy agents. Valproic acid, an antiepileptic drug, has been found to be a powerful HDAC inhibitor [162]. Gottlicher et al. demonstrated that valproic acid could trigger differentiation of transformed hematopoietic progenitor cells and leukemic blasts from AML patients [162]. Furthermore, Travaglini et al. found that valproic acid was able to epigenetically reprogram breast cancer cells into a more “physiologic” phenotype, thus improving sensitivity to other forms of breast cancer therapy [163]. In addition, entinostat, a selective inhibitor of class I HDACs, was recently reported to reverse EMT phenotype and decrease the population of tumor-initiating cells in triple-negative breast cancer (TNBC) [164]. These tumor-initiating cells possessed CSC properties and were responsible for driving metastasis and drug resistance in TNBC, thus contributing to poor patient prognosis. Hence, this study demonstrated the utility of HDAC inhibitors in preventing CSC invasiveness and tumor metastasis. Overall, these studies demonstrate the potential use of epigenetic modulators towards the differentiation and therapeutic sensitization of CSCs.

#### Targeting histone methylation – Histone methyltransferase (HMT) inhibitors

A class of enzymes called histone lysine methyltransferases (HKMTs) mediate the addition of a methyl group to the nitrogen atom of the lysine side chain [165]. Despite catalysing a common chemical reaction, this family of HKMTs demonstrate large structural diversity of its active sites, allowing these enzymes to have high substrate specificity [127]. For example, DOT1L (KMT4) is a unique HKMT as it is currently the only known enzyme that methylates lysine 79 of histone H3 (H3K79) [166]. Similarly, methylation of H3K27 is only mediated by the catalytic subunit EZH2 (KMT6) of PRC2 [127]. In contrast, some methylation marks can be catalysed by several proteins, such as H3K9 methylation. These post-translational methylation of histones have important roles in regulation of gene expression, differentiation, DNA damage repair as well as in tumorigenesis [167, 168]. Aberrant histone methylation can be due to gene mutations, over-expression or deregulated control of epigenetic modulatory enzymes involved. Thus, HKMTs are potential therapeutic targets, and the structural differences between members of the family also enable greater selectivity in inhibition of these proteins by small molecule compounds [169].

HKMT inhibitors have only recently gained more attention as cancer therapeutics, resulting in a rapidly increasing number of these small molecule inhibitors being developed [170–172]. In fact, several DOT1 like histone H3K79 methyltransferase (DOT1L) and EZH2 inhibitors have progressed to being tested in clinical trials as cancer interventions [173]. H3K79 methylation by DOT1L is associated with transcriptional activation of genes under its regulation [174, 175], and overexpression or aberrant DOT1L activity has been found in cancer, such as leukemia with mixed lineage leukemia (MLL) gene translocation. The MLL fusion protein can recruit DOT1L into a transcription complex, which subsequently methylates H3K79 [176–180]. This leads to dysregulation and overexpression of many MLL-target genes, including Homeobox A9 (HoxA9) and Meis homeobox 1 (Meis1), which are key regulators of hematopoietic stem cell differentiation that contributes to leukemogenesis [165]. Therefore, DOT1L is an attractive target for therapy, resulting in the first selective DOT1L inhibitor EPZ-4777 to be synthesised with anti-tumor effects against murine models of MLL-rearranged leukemia [181]. Further optimisation of the drug led to the development of EPZ-5676, the first HKMT inhibitor to enter clinical trials. This compound has been shown to be highly potent and selective for DOT1L. Treatment with EPZ-5676 in a MLL-rearranged leukemia xenograft model showed durable and complete tumor regression [182]. EPZ-5676 is currently under clinical studies (Phase I) for MLL-fusion leukemia, AML, MDS and myeloproliferative disorders.

EZH2 is a member of PRC2, along with proteins embryonic ectoderm development protein (EED) and SUZ12, and is responsible for catalysing H3K27 mono-, di- and tri-methylation [183–185]. Overexpression of EZH2 has been found in various cancers of the breast, lung, prostate and haematological malignancies [186–191], and is associated with poor disease prognosis. Studies have also shown the role of EZH2 deregulation in tumor progression, metastasis [192, 193] and maintenance of CSC self-renewal properties [194]. In glioblastoma multiforme (GBM), inhibition of EZH2 by S-adenosylhomocysteine hydrolase (SAH) inhibitor 3-deazaneplanocin A (DZNep) was able to reduce self-renewal and tumor-initiating capabilities of GBM CSCs in vivo via affecting transcriptional regulation of oncogene *MYC* [193]. However, DZNep affects methylation of other histone residues [195], leading to the development of more specific EZH2 inhibitors. The earliest SAM-competitive and selective EZH2 inhibitor to advance into clinical trials for treatment of rhabdoid tumors and lymphomas is EPZ-6438 (E7438) [196, 197]. A more recent drug, GSK2816126 (GSK126) has also entered clinical studies for relapsed/refractory diffuse large B-cell lymphoma (DLBCL), multiple myeloma and transformed follicular lymphoma [198, 199]. Both drugs have shown high potency and selectivity in inhibiting tumor growth in preclinical studies [197, 198, 200].

H3K9 methyltransferases, such as euchromatic histone lysine methyltransferase 2 (G9a/EHMT2) and euchromatic histone lysine methyltransferase 1 (GLP/EHMT1), catalyse mono- and di-methylation of the lysine residue, while tri-methylation of H3K9 is mediated by Suppressor of variegation 3–9 homolog 1 (SUV39H1) and Suppressor of variegation 3–9 homolog 2 (SUV39H2) [201]. Upregulation of G9a activity has been linked to several types of cancer, including ovarian, lung, liver and bladder cancers [202–208]. Hence, several substrate-competitive inhibitors of these HKMTs have been developed. BIX-01294 is the first specific inhibitor of G9a and GLP, and studies have reported its ability to decrease H3K9me2 levels in mammalian cells [209–211]. Kim et al. reported that BIX-01294 was able to induce cell death in colon and breast cancer cells via EHMT dysfunction [212]. However, due to the increased toxicity levels of BIX-01294 at higher concentrations, the use of this drug is limited. This led to the recent development of a more potent, specific and selective EHMT inhibitor, UNC0638 that was found to decrease local H3K9me2 and DNA methylation levels [213]. Further development generated UNC0642, which possessed better pharmacokinetic properties and higher efficacy in inhibiting colony formation ability of pancreatic adenocarcinoma cells [214].

Methylation of H3K9 by SUV39H1 is associated with silencing of tumor suppressor genes, including E-cadherin and p15INK4B, in AML [215]. Overexpression of SUV39H1 has also been correlated with poor prognosis in multiple myeloma patients [216]. Treatment of multiple myeloma cells with chaetocin, a small molecule inhibitor of SUV39H1 showed anti-tumor effects at low doses of the drug [216]. Similarly, chaetocin was found to decrease H3K9me3 levels and induce differentiation of AML cells at non-toxic doses [217]. Furthermore, chaetocin was able to repress cell proliferation and induce apoptosis in hepatocellular carcinoma (HCC) cultures and xenografts [218], implying a potential tumorigenic role of EHMTs in HCC progression and development.

#### Targeting histone demethylation - Histone demethylase (HDM) inhibitors

Methylation of lysine on histones is also regulated by histone lysine demethylases (KDMs). This group of epigenetic erasers function in removing the methyl groups from lysine side chains on histones [219, 220]. As proper functioning of both HKMTs and KDMs is required to maintain stable histone methylation levels, small molecule inhibitors have also been developed to target KDMs. KDMs can be grouped into two families - the lysine-specific demethylase (LSD) family and Jumonji domain-containing (JmjC) family [221]. The LSD family are flavin adenine dinucleotide (FAD)-dependent amine oxidase that demethylates mono- and di-methyl lysine residues, while JmjC enzymes utilise 2-oxoglutarate and iron to oxidatively release methyl groups from all three methylation states at lysine residues [172, 222].

Upregulated expression of LSD1 (KDM1A) has been found in various human cancers, including AML, ovarian, lung, bladder and colorectal cancers [223–225]. Hence, small molecule inhibitors of LSD1 that target the enzyme cofactor FAD have been developed, the first of which is tranylcypromine [226]. Further studies have led to the synthesis of more selective derivatives of tranylcypromine, such as ORY-1001 [227] and GSK2879552 [228]. They function by irreversibly changing FAD, leading to the formation of a tetracyclic adduct [229]. LSD1 is important for normal hematopoiesis; loss of LSD1 has been found to inhibit differentiation and impair hematopoiesis [230]. This suggests a potential role of aberrant LSD1 activity in affecting stemness properties in tumor cells. The inhibitor ORY-1001 has been shown to decrease the population of AML stem cells and improve survival of mice with acute lymphoblastic leukemia (ALL) in preclinical studies [227, 228]. GSK2879552 has also been found to influence differentiation in small cell lung cancer (SCLC) [231]. These compounds are currently in phase 1 studies for relapsed or refractory AML (ORY-1001) and SCLC (GSK2879552).

Similarly, JmjC demethylases are amenable to pharmacological intervention as well. Ubiquitously transcribed tetratricopeptide repeat X chromosome (UTX), also known as KDM6A, is responsible for demethylating H3K27 [232–234], and loss of UTX activity has been found in multiple human malignancies, including multiple myeloma, esophageal squamous cell carcinoma and renal carcinoma [166]. However, no inhibitors of JmjC enzymes have advanced beyond biochemical studies [127]. Nevertheless, as UTX is a component of the mixed lineage leukemia protein 2 (MLL2) H3K4 methyltransferase complex, and interacts with SWI/SNF chromatin remodelling complex [235–237], it is still an important epigenetic target and its role in epigenetic modulation still warrants further study.

#### Targeting epigenetic readers – BET inhibitors

While epigenetic modulatory enzymes are obvious targets for therapy, epigenetic readers are also important components of the epigenetic machinery as they directly or indirectly regulate gene expression. One such group of readers called bromodomain and extra-terminal (BET) proteins modulate gene expression by recognising acetylated histones. Increased BET activities have been associated with NUT midline carcinoma (NMC), glioblastoma and various haematological malignancies, through aberrant transcription of disease-associated genes and oncogenes such as *MYC* [238]. Hence, BET proteins appear to be attractive therapeutic targets for controlling dysregulated gene expression.

JQ1 is a selective BET inhibitor of BRD family of proteins, including Bromodomain-containing protein 4 (BRD4) [239]. In preclinical studies, JQ1 was able to cause tumor regression in NMC mouse models, inhibit proliferation, induce apoptosis and differentiation in cancer cells [239–242]. Another BET inhibitor, I-BET762 (GSK525762A), functions by binding to the acetyl-binding pocket of BET proteins [243, 244]. Studies have shown that I-BET762 treatment was able to induce terminal differentiation of patient-derived malignant cells [245] and activate apoptosis in neuroblastoma and prostate cancer via inhibition of Myc-driven pathways [246, 247]. This compound is currently in phase I trials for solid tumors and relapsed or refractory haematological cancers.

OTX015 is another BET inhibitor that has progressed into clinical trials for various haematological malignancies. This compound has been found to possess anti-proliferative effects via directly influencing *MYC* expression and activity [248, 249]. Similarly, CPI-0610 has also entered clinical testing for lymphoma, multiple myeloma and myelodysplastic or myeloproliferative neoplasms. I-BET151 is a pan-BET inhibitor, similar to JQ1, and has been found to block proliferation and induce apoptosis in myeloma cells via repressing Myc activity [250]. Antitumor effects have also been observed in NMC, MLL, ALL, lung cancer and brain cancer [238].

#### Combination therapy with epigenetic modulators

While epigenetic drugs have been tested preclinically and clinically as single agents, further studies have revealed the increased efficacy of these drugs when used in combination with other therapies. One common combination of different epigenetic therapies is that of DNMT and HDAC inhibitors. Pathania et al. reported that combining azacitidine (DNMT inhibitor) and butyrate (HDAC inhibitor) was capable of significantly decreasing breast cancer CSC population [251]. In addition, combination of azacitidine and HDAC inhibitor entinostat at low doses in a phase I/II clinical trial showed sustained and favourable responses in treatment-resistant non-small cell lung cancer (NSCLC) patients [252]. Azacitidine and valproic acid co-treatment was also able to promote tumor regression in *Patched* mutant mouse models of medulloblastoma [253]. Besides DNMT-HDAC inhibitor combination therapy, studies have demonstrated synergistic effects of other epigenetic drug combinations. For example, inhibiting both EZH2 and G9a histone methyltransferases showed greater efficacy in blocking cell proliferation as compared to single drug treatment [254]. Furthermore, the DOT1L inhibitor EPZ-5676 could interact synergistically with DNA hypomethylating agents, such as azacitidine and decitabine, in MLL-rearranged leukemia cells [255].

In recent years, an increasing number of studies have reported the use of epigenetic drugs in combination with conventional chemotherapeutics, with underlying mechanisms of re-sensitising resistant CSCs to drug treatment, or to prime cancer cells for subsequent therapies [134, 256]. For example, low doses of SGI-110 (DNMT inhibitor) was found to drive ovarian CSCs towards a more differentiated phenotype and sensitise them to platinum treatment [125]. DOT1L inhibitor EPZ-5676 was also able to establish a chromatin state that enhanced the anti-tumor effects of cytarabine or daunorubicin in MLL-rearranged leukemia [255]. Moreover, pre-treatment with azacitidine was demonstrated to prime colon cancer cell lines to irinotecan therapy [257]. Indeed, various combinations have been tested in clinical trials with promising results on drug response and anti-tumor efficacy [258–261]. In addition to drug combination synergy, the method of delivery could also improve response to therapy. A recent paper by Li et al. showed that encapsulating decitabine and doxorubicin in nanoparticles was able to better target breast CSCs and inhibit tumor growth [262].

The use of immunotherapy in cancer has made significant progress over the past two decades, with several immunotherapy drugs being approved by the FDA for the treatment of cancer. These drugs function to overcome the mechanisms of immune tolerance that are employed by cancer cells to evade or limit the body’s immune response. These mechanisms include changes in antigen processing and presentation, creation of an immunosuppressive microenvironment, induction of T-cell death and activation of negative immune regulatory pathways [263]. One key receptor involved in the immunoinhibitory pathways is the cytotoxic T-lymphocyte-associated protein 4 (CTLA-4), which is expressed on the surface of immune cells and acts as an immune checkpoint. Studies have shown that targeting CTLA-4 receptor induced favourable responses in patients with advanced melanoma [264], and the FDA-approved CTLA-4 inhibitor, Ipilimumab, is now in clinical trials for prostate and lung cancers. Another immune checkpoint involved in tumor immune-resistance is the interaction between programmed cell death-1 (PD-1) and programmed death-ligand 1 (PD-L1) [265]. Specific targeting of PD-1 and PD-L1 has been clinically shown to be very effective in treatment of metastatic cancers and melanomas [266, 267].

However, as most of these immunotherapy strategies are mainly targeted at bulk tumors, which contain more differentiated cells with “differentiation antigens” [268], CSCs (which have a different set of tumor antigens) would not be successfully eradicated. Hence, more effective targeting of the CSC population can be achieved via CSC-specific immunologic approaches, or by combining immunotherapy with epigenetic therapies that induce CSC differentiation and alter surface protein expression. The latter approach would likely improve the overall antitumor efficacy as both CSC and bulk tumor populations can be targeted simultaneously. For instance, the use of DNA hypomethylating agent (5-aza-2^’^-deoxycytidine) in combination with anti-CTLA-4 monoclonal antibody in syngeneic transplantable murine models demonstrated significant reduction in tumor volumes as compared to single agent treatment alone [269]. The improved efficacy of this combination was attributed to the increased CD3+ T-cell infiltration in the combination cohort tumors and a sustained expression of cancer antigens and MHC proteins due to promoter demethylation. Furthermore, combinatorial drug treatment with immune checkpoint inhibitors (anti-CTLA-4 and anti-PD-1) and epigenetic modulators (5-azacytidine and Entinostat) showed remarkable eradication of CT26 colorectal tumors and 4 T1 mammary tumors in more than 80% of the tumor-bearing mice [270]. Importantly, 4 T1 tumor-bearing mice that were given combinatorial treatment did not develop metastases as compared to mice given single agent treatment. These findings demonstrate that epigenetic drugs in combination with immunotherapy can enhance the reversal of immune tolerance in cancer cells, including CSCs.

Another way in which cancer cells evade cytotoxic T-cells is by down-regulating human leukocyte antigen (HLA) to avoid tumor antigen presentation [134]. Hypermethylation of HLA promoters was frequently observed in gastric cancer and esophageal squamous cell cancers [271, 272]. Treatment with DNMT and HDAC inhibitors were found to be capable of reversing this hypermethylation and increasing HLA expression [272–275], thus priming these cells for immunotherapy. In addition, Li et al. showed that azacitidine treatment was able to enhance immunomodulatory pathways, such as antigen processing/presentation and interferon signaling, in breast, colorectal and ovarian cancers [276]. These preclinical data highlight the promising potential of combining epigenetic and immunotherapies in improving cancer treatment efficacy, which will be verified in several ongoing clinical trials.

## Conclusion

Our understanding of cancer has changed over the last decade with the advances in sequencing technologies and the deciphering of the human genome. It is now clear to us that the tumor genome is complex and heterogeneous and that tumors do not arise from a single clone with a single tumor genome. We have discussed several important aspects and examples of how epigenetic deregulation may drive or promote tumorigenesis and metastasis by alteration of key transcriptomic programs and signaling pathways, especially in CSCs. More importantly, we have provided several evidences that these epigenetic modifiers are targetable and many of these epigenetic modulating drugs have entered clinical trials, and some including azacitidine, decitabine, vorinostat and romidepsin have been approved for various indications by the FDA. We believe that the success of these epigenetic therapeutic trials will provide a promising path to follow.

## Abbreviations

ABC: ATP-binding cassette
ABCG2: ATP-binding cassette sub-family G member 2
ALL: Acute lymphoblastic leukemia
AML: Acute myeloid leukemia
AML1-ETO: Acute myeloid leukemia protein 1 Eight twenty-one protein
APC: Adenomatous polyposis coli
ASCL1: Achaete-scute family BHLH transcription factor 1
BCC: Basal cell carcinoma
BCL2: B-cell lymphoma 2
BET: Bromodomain and extra-terminal
Bmi1: B-lymphoma Mo-MLV insertion region 1 homolog
CK1: Casein kinase 1
CK19: Cytokeratin 19
CSC: Cancer stem cells
DACT3: Polycomb repressor complex 2, PCR2 Dishevelled-binding antagonist of beta-catenin 3
*DKK1*: Dickkopf-related protein 1
DNMTs: DNA methyltransferases
DZNep: 3-deazaneplanocin A
ECM: Extracellular matrix
EED: Embryonic ectoderm development protein
EMT: Epithelial-to-mesenchymal transition
EpCAM: Epithelial cell adhesion molecule
ESCs: Embryonic stem cells
EZH2: Enhancer of zeste homolog 2
FAD: Flavin adenine dinucleotide
G9a/EHMT2: Euchromatic histone lysine methyltransferase 2
GBM: Glioblastoma multiforme
GLP/EHMT1: Euchromatic histone lysine methyltransferase 1
GNPs: Granule neuron progenitors/precursors
GSK-3β: Glycogen synthase kinase 3 beta
H3K27: Histone H3 lysine 27
H3K36: Histone H3 lysine 36
H3K4: Histone H3 lysine 4
H3K79: Histone H3 lysine 79
H3K9: Histone H3 lysine 9
H4K20: Histone H4 lysine 20
HAT: Histone acetyltransferases
HCC: Hepatocellular carcinoma
HDAC: Histone deacetylase
Hh: Hedgehog
HKMT: Histone lysine methyltransferase
HoxA9: Homeobox A9
ICC: Intrahepatic cholangiocarcinoma
JmjC: Jumonji domain-containing (JmjC)
KDM: Histone lysine demethylase
Kif7: Kinesin family member 7
LDR5/6: Low density lipoprotein receptor-related protein 5/6
LSD: Lysine-specific demethylase
MDR1: Multidrug resistance protein 1
Meis1: Meis homeobox 1
miRNAs: Micro RNAs
MLL: mixed lineage leukemia
MLL2: Mixed lineage leukemia protein 2
MRP1: Multidrug resistance-associated protein 1
NF-κB: Nuclear factor kappa b
NICD: Notch intracellular domain
NKD1: Naked cuticle homolog 1
NMC: NUT midline carcinoma
NSCLC: non-small cell lung cancer
NSCs: Neural stem cells
OCT4: Octamer-binding transcription factor 4
PML-RARα: Promyelocytic leukemia protein retinoic acid receptor alpha
PTCH1: Patched receptor
RBPJ-κ: Recombination signal binding protein for immunoglobulin kappa J region
SAH: S-adenosylhomocysteine hydrolase
SAM: S-adenosyl methionine
SFRP-1: Secreted frizzled-related protein 1
Shh: Sonic hedgehog ligand
SirT1: Sirtuin 1
SMO: Smoothened
SMRT: Nuclear receptor co-repressor 2
SNAIL: Snail family zinc finger 1
SP: Side population
STRAP: Serine-threonine kinase receptor-associated protein
SUFU: Suppressor of fused homolog
SUV39H1: Suppressor of variegation 3–9 homolog 1
SUV39H2: Suppressor of variegation 3–9 homolog 2
Suz12: Suppressor of zeste 12 protein homolog
TCF/LEF: T-cell factor/lymphoid enhancer factor
TGF-β: Transforming growth factor-β
TNBC: Triple-negative breast cancer
TWIST1: Twist-related protein 1
UTX: Ubiquitously transcribed tetratricopeptide repeat, X chromosome (UTX)
WIF-1: Wnt inhibitory factor 1
ZEB1: Zinc finger E-box-binding homeobox 1
ZEB2: Zinc finger E-box-binding homeobox 2
